# Faecal corticosterone metabolite concentrations are not a good predictor of habitat suitability for common gartersnakes

**DOI:** 10.1093/conphys/cov047

**Published:** 2015-10-20

**Authors:** William D Halliday, Kathleen M Gilmour, Gabriel Blouin-Demers

**Affiliations:** Department of Biology, University of Ottawa, 30 Marie Curie, Ottawa, Ontario, Canada K1N 6N5

**Keywords:** Fitness, habitat quality, habitat selection, reproductive output, stress physiology, *Thamnophis sirtalis*

## Abstract

Measuring habitat suitability is important in conservation and in wildlife management. Measuring the abundance or presence–absence of a species in various habitats is not sufficient to measure habitat suitability because these metrics can be poor predictors of population success. Therefore, having some measure of population success is essential in assessing habitat suitability, but estimating population success is difficult. Identifying suitable proxies for population success could thus be beneficial. We examined whether faecal corticosterone metabolite (fCM) concentrations could be used as a proxy for habitat suitability in common gartersnakes (*Thamnophis sirtalis*). We conducted a validation study and confirmed that fCM concentrations indeed reflect circulating corticosterone concentrations. We estimated abundance, reproductive output and growth rate of gartersnakes in field and in forest habitat and we also measured fCM concentrations of gartersnakes from these same habitats. Common gartersnakes were more abundant and had higher reproductive outputs and higher growth rates in field habitat than in forest habitat, but fCM concentrations did not differ between the same two habitats. Our results suggest either that fCM concentrations are not a useful metric of habitat suitability in common gartersnakes or that the difference in suitability between the two habitats was too small to induce changes in fCM concentrations. Incorporating fitness metrics in estimates of habitat suitability is important, but these metrics of fitness have to be sensitive enough to vary between habitats.

## Teaser

We demonstrate that abundance, reproductive output, and growth rate of Common Gartersnakes living in field and forest habitats are good metrics of habitat suitability, whereas stress hormone levels show no difference in suitability between habitats.

## Introduction

Habitat loss is one of the greatest threats to global biodiversity (Sala *et al*., 2000). For this reason, estimating habitat suitability is an integral part of wildlife management and conservation, allowing for better-informed decisions about what habitats must be preserved to maintain species of conservation concern (Morris, 2003). Traditionally, habitat suitability has been estimated via presence–absence or abundance data in various habitats (Guisan and Zimmermann, 2000). Abundance may not always be a good metric of habitat suitability (Van Horne, 1983); animals are not always most abundant where the population growth rate (the ultimate measure of population success) is the highest. Moreover, habitat occupancy does not always imply habitat quality (Johnson, 2007).

Ideal despotic distributions (Fretwell and Lucas, 1969) are a clear example of a mismatch between abundance and population success. Under an ideal despotic distribution, dominant individuals occupy high-quality habitats and exclude subordinates from such habitats. Therefore, a few dominant individuals experience high fitness in high-quality habitats, whereas many subordinates experience low fitness in lower-quality habitats. For example, dominant European jays live in high-quality older forests at low densities and achieve high fitness, whereas subordinate jays live in poorer-quality habitats at high densities and have lower fitness owing to higher predation (Andrén, 1990). Likewise, a few large, dominant brook trout occupy high-quality pools and exclude many smaller subordinates that then live in lower-quality riffle segments of streams (Knight *et al*., 2008). Under an ideal despotic distribution, therefore, high density does not imply high population success in a habitat.

Habitat occupancy also can be a misleading metric of habitat quality because metapopulations often have source–sink dynamics (Pulliam, 1988). In a source–sink system, populations in the source habitat increase in size, whereas populations in the sinks decrease in size; dispersal from the source to the sink maintains the population in the sink habitat. For example, Caribbean spiny lobsters occur in source habitats where spawning takes place and where nurseries are located, but local hydrodynamics cause larvae to disperse passively into sink habitats that are not suitable for spawning or as nurseries (Lipcius *et al*., 1997). Source–sink systems can also be maintained via active dispersal. Checkerspot butterflies actively disperse between habitats throughout the season, and populations in clearing habitats late in the season decrease in size owing to senescence of the host plants (Boughton, 1999). On the contrary, butterfly populations in outcrop habitat late in the season increase in size because the host plant in outcrops does not senesce as early as the host plant in clearings. The outcrop habitat acts as a source for the low-quality clearing habitat. Finally, ‘ecological traps’ have been documented, in which animals prefer habitats where their reproductive success is low (Gates and Gysel, 1978; Battin, 2004; Weldon and Haddad, 2005).

Ideal despotic distributions, source–sink systems and ecological traps exemplify why abundance and occupancy are not necessarily useful measures of habitat suitability. Ecologists have been advocating the use of measures other than abundance to estimate habitat suitability for >30 years (Van Horne, 1983; Homyack, 2010; Cooke *et al*., 2013), but this advice is still often not heeded (Johnson, 2007). Although ultimate measures of mean population fitness are difficult to obtain, especially in long-lived species, proximate measures of mean population fitness can be more easily obtained. Such proximate measures include individual growth rate (Halpin, 2000; Manderson *et al*., 2002), fecundity (Morris, 1989; Halliday and Blouin-Demers, 2014; Halliday *et al*., 2015) or physiological metrics, such as field metabolic rate or concentrations of glucocorticoid stress hormones (Homyack, 2010). For example, corticosterone concentrations of salamanders differ between landscapes with varying human disturbance and in natural vs. anthropogenic habitats (Homan *et al*., 2003). Corticosterone concentrations also differ among birds living in forests with varying fragmentation (Soursa *et al*., 2003). Snakes living near roads tend to have a reduced corticosterone stress response compared with snakes living away from roads (Owens *et al*., 2014). Increased predation (Boonstra *et al*., 1998) and increased population density in a habitat can also increase stress levels (Montero *et al*., 1999). In some circumstances, therefore, corticosterone concentrations may be useful indicators of habitat quality.

Corticosterone concentrations are sometimes too variable to be a good indicator of habitat suitability. For example, different levels of human disturbance had no effect on corticosterone concentrations in eastern hellbenders (Hopkins and DuRant, 2011). Corticosterone concentrations may also vary between seasons, and concentrations can differ between habitats in one season but not in another (Marra and Holberton, 1998). Moreover, the relationship between glucocorticoid concentrations and fitness is not consistent across taxa (Bonier *et al*., 2009). Thus, it remains unclear whether glucocorticoid concentrations can be a reliable indicator of habitat suitability.

In the present study, we evaluated the usefulness of faecal corticosterone metabolite (fCM) concentrations as a proximate measure of habitat suitability for common gartersnakes (*Thamnophis sirtalis*) living in field vs. forest habitat. We conducted a validation study and confirmed that fCM concentrations indeed reflect circulating corticosterone concentrations. We compared baseline fCM concentrations with abundance, individual growth rate and reproductive output of gartersnakes living in each habitat. Field and forest have very different thermal regimens; field is thermally superior to forest for snakes in our study area (Blouin-Demers and Weatherhead, 2001; Row and Blouin-Demers, 2006; Blouin-Demers and Weatherhead, 2008). For this reason, energy acquisition and assimilation should be higher in field than in forest, which should lead to higher individual growth rates and reproductive success in field than in forest. If fCM concentration is an appropriate proxy for habitat quality in garter snakes, it should reflect the inter-habitat patterns in the other proximate measures of fitness. If fCM concentration is unaffected by thermal or energetic differences, however, it will not vary between habitats. We included body temperature available to snakes to account for its potential direct effect on fCM (Cree *et al*., 2003).

## Materials and methods

### Abundance estimates

We monitored the habitat use of common gartersnakes between field and forest habitats at Queen's University Biological Station, Ontario, Canada (44°33′N, 76°21′W). We set up five pairs of 2500 m^2^ plots in adjacent fields and forests and placed eight plywood coverboards (360 cm^2^) on each plot (Halliday and Blouin-Demers, 2015). We systematically walked back and forth across each plot and checked under all of the coverboards twice per day over three consecutive days every 2 weeks from 5 May to 16 July 2013. We uniquely marked each individual gartersnake using ventral scale branding (Winne *et al*., 2006). We counted the number of different individual gartersnakes caught in each field and forest grid throughout the study and compared abundance in field vs. (adjacent) forest habitat using a paired *t* test in R (package, stats; function, t.test; R Core Team, 2014).

### Fitness and faecal corticosterone “metabolite estimates

We examined the growth rate, reproductive output and fCM concentrations of 20 female common gartersnakes in enclosures in Pontiac County, Québec, Canada (45°29′N, 75°55′W). We built 2.67 m × 2.67 m × 1.33 m frames out of lumber and created walls for the enclosure by attaching polyethylene vapour barrier to the frames. We buried the bottom 10 cm of the walls of the enclosures in the ground to prevent snakes from escaping. We built six of these enclosures in field habitat and an additional six in forest habitat. Each enclosure contained a 60 cm × 60 cm plywood coverboard for shelter. In late May 2014, we placed 10 females in forest enclosures and the other 10 females in the field enclosures; snakes were housed either in pairs or on their own. We captured all female snakes for this experiment in fields and wetlands near Ottawa, Ontario (*n* = 3) and close to our enclosures in Québec (*n* = 17). In the laboratory, before putting females in the enclosures, we gave each female the opportunity to mate with three or four males collected from the same area. We observed every female mate during these encounters. We fed half of the snakes in each habitat two large earthworms per week and we fed the other half of the snakes four large earthworms per week to determine whether fCM concentrations could be an indicator of habitat quality only with certain caloric intake levels. We systematically placed females in each food and habitat treatment combination based on their body size to ensure that each combination had similar distributions of body sizes, because body size may influence growth, reproductive frequency and reproductive output. We measured the body temperature of each snake with an infrared thermometer immediately before feeding it. We measured the mass and snout–vent length (SVL) of each snake once per week and also attempted to collect a faecal sample from each snake once per week. Faecal samples were stored in individual sealed tubes and were kept on ice until they were returned to the laboratory, where they were frozen at −80°C until they were processed. Snakes started giving birth in late August and finished by early September. We removed all snakes from the enclosures after the snakes gave birth and returned all mothers and their offspring to their point of capture.

We extracted fCMs from faecal pellets following the methods of Berkvens *et al*. (2013). Briefly, we placed thawed and homogenized (using a metal spatula cleaned with 80% methanol) faeces in 2.0 ml tubes with 80% methanol at a ratio of 0.1 g of faeces per 1.0 ml of methanol. We agitated this mixture on a magnetic stir plate at room temperature for 18 h. Following this period, we centrifuged each tube at 210***g*** for 10 min, decanted the liquid (henceforth referred to as the extract) into a new tube and stored the extract at −80°C until we measured fCM content by commercial radioimmunoassay (RIA). The extraction efficiency for this procedure was estimated by splitting a sample in two, spiking one half of the sample with a known amount of corticosterone, measuring corticosterone/fCM content in each half and comparing recovered corticosterone with the amount added, for six independent samples; using this method, extraction efficiency was 50%. This value is relatively low compared with ∼90% extraction efficiency for the faeces of ground squirrels (*Spermophilus beldingi*; Mateo and Cavigelli, 2005), but on par with the 49% extraction efficiency reported for faeces of African house snakes (*Lamprophis fuliginosus*; Berkvens *et al*., 2013). The present study is the first, to our knowledge, in which fCM concentrations were measured in faecal samples of gartersnakes. For this reason, we conducted a validation experiment (see online supplementary material Appendix S1). Briefly, we compared fCM concentrations with plasma corticosterone concentrations in gartersnakes before and after they were fed corticosterone-injected earthworms. We demonstrated that fCM concentrations reflect plasma corticosterone concentrations.

We measured the fCM content in each sample using a corticosterone double-antibody RIA kit (MP Biomedicals, Santa Fe, CA, USA), which was previously used by Roberts *et al*. (2009) to measure corticosterone in the plasma of *T. elegans*. We followed the directions of the RIA kit, except that we mixed 20 μl of extract in 1 ml of steroid diluent rather than the suggested 10 μl of sample in 2 ml of steroid diluent, to take into account the low concentrations of corticosterone metabolites in the samples. All experimental samples were assayed together in a single assay, for which intra-assay variability was 8.7%. In addition, serial dilution of extracted fCM samples yielded a displacement curve parallel to the corticosterone standard curve (online supplementary material Appendix S1).

We calculated the change in mass of snakes as the difference in the mass of a snake between the beginning and the end of the experiment. Likewise, we calculated the change in SVL of snakes from the beginning to the end of the experiment. We compared changes in mass and SVL of snakes in field vs. forest habitat using general linear models in R (package, stats; function, lm; R Core Team, 2014), with habitat, food treatment and their interaction as independent variables. We also included the number of weeks that a snake was in the experiment to control for differences in time to grow (a few snakes escaped the enclosure or were eaten by predators before the end of the experiment) and the starting mass or SVL of the individual to control for size-dependent differences in growth rate. We used bias-corrected Akaike's information criteria (package, qpcR; function, AICc; Spiess, 2014) for model selection and considered the model with the lowest AICc to be the best model. Models within 2 AICc units of the best model were considered to be competing models (Bozdogan, 1987), and we used model averaging between competing models to determine the parameter estimates for the final model (Burnham and Anderson, 2002).

We examined fCM concentrations using mixed-effects models in R (package, nlme; function, lme; Pinheiro *et al*., 2014), with habitat, food treatment and their interaction as fixed effects and with snake ID nested within week into the experiment as random effects. We again used bias-corrected Akaike's information criteria for model selection. We also examined how fCM concentrations were affected by body temperature using another mixed-effects model with the same random effects. We used the mean body temperature (i.e. mean of the four temperature measurements taken on the four feeding days) of each snake during the week preceding the collection of the faecal sample because the corticosterone metabolites found in faeces should be a function of the corticosterone in the plasma on previous days during which digestion occurred.

## Results

**Figure 1:**
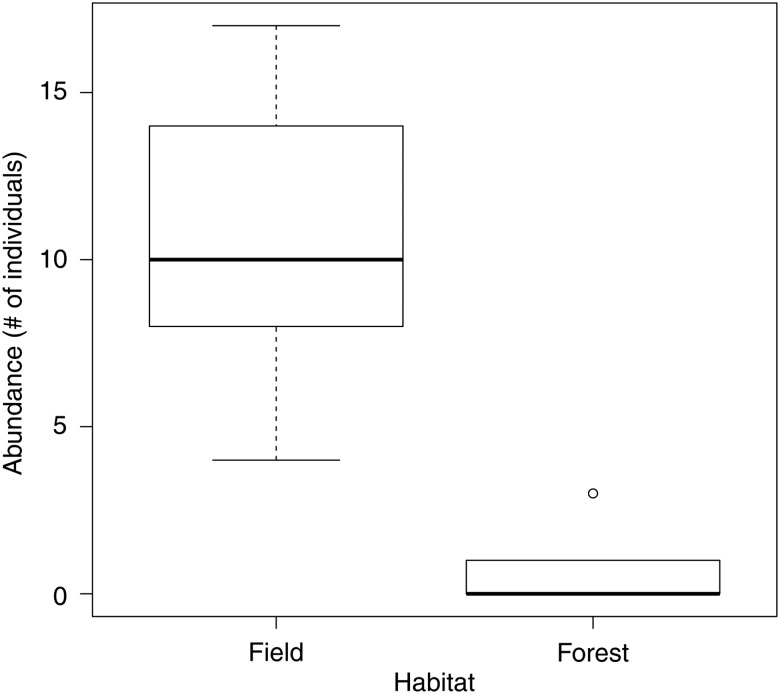
Abundance estimates of common gartersnakes (*Thamnophis sirtalis*) in field and in forest habitat at the Queen's University Biological Station in Ontario, Canada from May to July 2013. The box represents the interquartile range, the line within the box is the median, and the whiskers represent the 95% confidence interval. Points represent outliers outside the 95% confidence interval.

**Figure 2:**
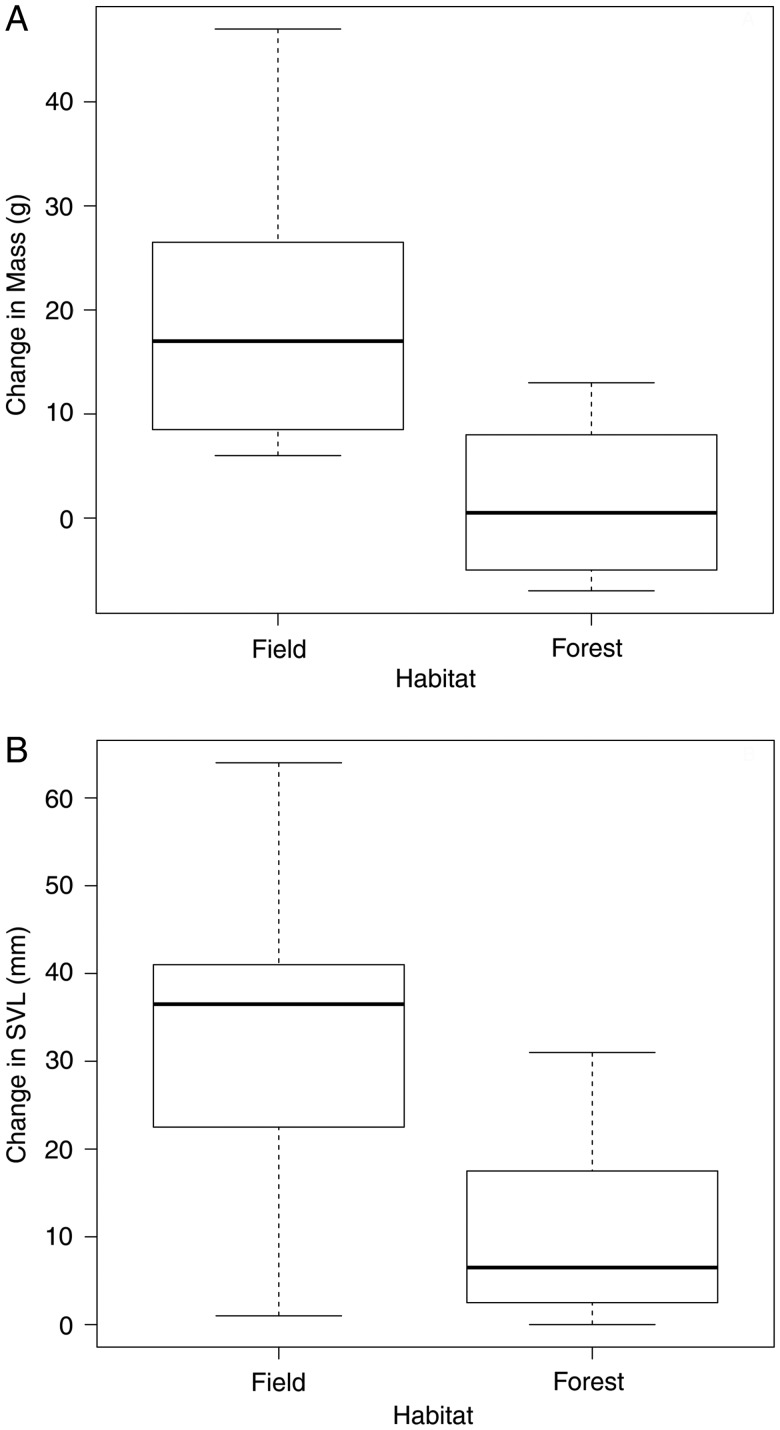
Change in mass (**A**) and in snout–vent length (SVL; **B**) of female common gartersnakes (*T. sirtalis*) living in enclosures in field and in forest habitat in Pontiac County, Québec, Canada from May to August 2014. The box represents the interquartile range, the line within the box is the median, and the whiskers represent the 95% confidence interval.

Common gartersnakes demonstrated a strong preference for field over forest habitat (mean abundance in field ± SEM = 10.6 ± 2.3; forest = 0.8 ± 0.6; mean difference = 9.8; *t*_4_ = 5.01, *P* < 0.01; Fig. 1). Four of the 10 female snakes in the field enclosures gave birth (average litter size = 9 ± 1 offspring), whereas no female snakes in the forest gave birth (*F*_14_ = 6.07, *P* = 0.03). Snakes in the field grew more in terms of both mass (field = 20 ± 5 g; forest = 2 ± 3 g; *t*_14_ = 3.16, *P* < 0.01; *r*^2^ = 0.42; Table 1 and Fig. 2A) and SVL (field = 33 ± 7 mm; forest = 11 ± 4 mm; *t*_13_ = 4.23, *P* < 0.01; *r*^2^ = 0.63; Table 2 and Fig. 2B) than snakes in the forest. Food treatment had no effect on mass gain (*t*_13_ = 0.81, *P* = 0.43) or on SVL increase (*t*_13_ = 0.41, *P* = 0.69).

**Table 1:** Model selection and final model output for linear models examining the change in mass of female common gartersnakes (*Thamnophis sirtalis*) living in enclosures in field and forest habitat with different food treatments

Model		*k*	AICc	ΔAICc
Mass ∼ Habitat		3	128.02	0.00
Mass ∼ Habitat + Start mass		4	130.25	2.23
Mass ∼ Habitat + Food		4	130.31	2.29
Mass ∼ Habitat + Start mass + Food + Habitat:Food		6	130.50	2.48
Mass ∼ Habitat + Start mass + Food + Habitat:Food + Weeks		7	138.48	10.46

Parameter	Estimate	SE	*t*	*P*-value
Intercept	19.63	4.02	4.88	<0.01
Habitat (forest)	−18.00	5.69	3.16	<0.01

Abbreviations: AICc is the bias-corrected Akaike's information criterion value; ΔAICc is the difference in AICc between each model and the model with the lowest AICc; and *k* is the number of parameters in the model.

**Table 2:** Model selection and final model output for linear models examining the change in snout–vent length of female common gartersnakes (*T. sirtalis*) living in enclosures in field and forest habitat with different food treatments

Model		*k*	AICc	ΔAICc
SVL ∼ Habitat + Start SVL		4	130.91	0.00
SVL ∼ Habitat + Start SVL + Food + Habitat:Food		6	132.37	1.44^a^
SVL ∼ Habitat + Start SVL + Food		5	132.69	1.78^a^
SVL ∼ Habitat + Start SVL + Food + Habitat:Food + Weeks		7	132.76	1.85^a^
SVL ∼ Habitat		3	137.48	6.87

Parameter	Estimate	SE	*t*	*P*-value
Intercept	124.50	32.03	4.04	<0.01
Habitat (forest)	−29.75	6.43	4.23	<0.01
Start SVL	−0.19	0.06	3.04	<0.01
Food	−7.18	6.70	0.41	0.69
Habitat:Food	15.11	12.81	1.31	0.22
Weeks	1.23	1.20	1.03	0.33

Abbreviations: AICc is the bias-corrected Akaike's information criterion value; ΔAICc is the difference in AICc between each model and the model with the lowest AICc; *k* is the number of parameters in the model; and SVL is the snout–vent length. ^a^Competing models that are within 2 AICc units of the best model. Estimates in the final model output are based on model averaging between competing models.

**Figure 3:**
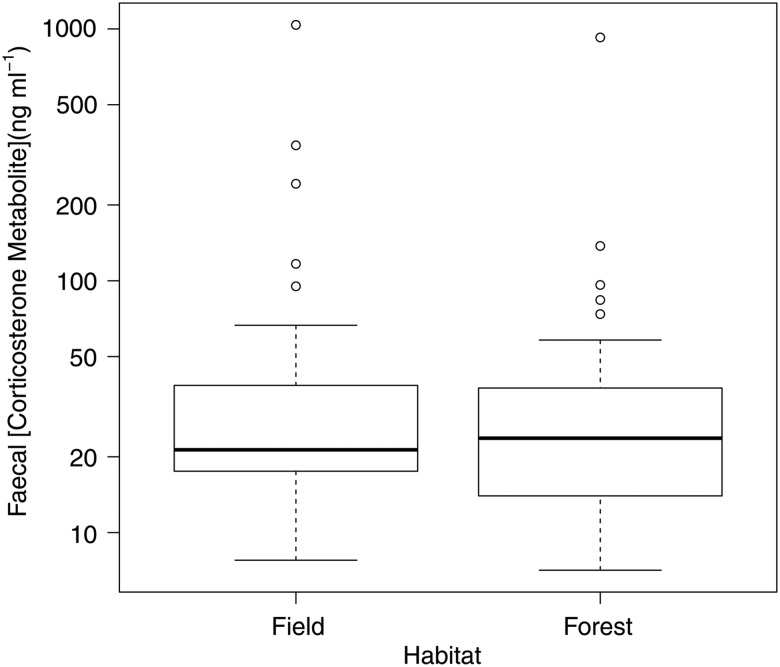
Faecal corticosterone metabolite concentrations of female common gartersnakes (*T. sirtalis*) living in enclosures in field and in forest habitat in Pontiac County, Québec, Canada from May to August 2014. The box represents the interquartile range, the line within the box is the median, and the whiskers represent the 95% confidence interval. Points represent outliers outside the 95% confidence interval.

Snakes in field and in forest did not differ in fCM concentrations (field = 65.7 ± 25.0 ng ml^−1^; forest = 59.1 ± 27.5 ng ml^−1^; *t*_76_ = 0.54, *P* = 0.60; Table 3 and Fig. 3). Food treatment had no effect on fCM concentrations (*t*_58_ = 0.04, *P* = 0.97), nor were fCM concentrations affected by body temperature (slope = 0.002 ± 0.01 ng ml^−1^ fCM °C^−1^; *t*_58_ = 0.18, *P* = 0.86).

**Table 3:** Model selection for linear mixed-effects models examining the faecal corticosterone metabolite concentrations of female common gartersnakes (*T. sirtalis*) living in enclosures in field and forest habitat with different food treatments

Model	*k*	AICc	ΔAICc
fCM ∼ 1	4	53.66	0.00
fCM ∼ Habitat	5	58.17	4.51
fCM ∼ Temperature	5	63.23	9.57
fCM ∼ Habitat + Food	6	63.69	10.03
fCM ∼ Habitat + Food + SVL	7	74.32	20.66
fCM ∼ Habitat + Food + SVL + All interactions	11	113.22	59.56

Abbreviations: AICc is the bias-corrected Akaike's information criterion value; ΔAICc is the difference in AICc between each model and the model with the lowest AICc; fCM is the faecal corticosterone metabolite; and *k* is the number of parameters in the model. The final model contained no predictor variables.

## Discussion

To determine habitat suitability, wildlife managers and conservation biologists must use measures of success in different habitats to confirm that a habitat that is occupied also provides high mean population fitness. In this study, we compared fCM concentrations with abundance, individual growth rate and reproductive output of common gartersnakes in field and in forest habitats to test whether fCM concentrations could be a useful measure of habitat suitability. As predicted, abundance, growth rate and reproductive output of gartersnakes were higher in field than in forest habitat. However, fCM concentrations of female gartersnakes did not differ between habitats despite clear indications from our other measures of success that gartersnakes were doing better in field than in forest habitat. For instance, female snakes in the forest did not gain mass as the experiment progressed, whereas females in the field gained mass. It is possible that the fitness differences between habitats in our study were not sufficient to be reflected in fCM concentrations. However, the marked effects of habitat on our other metrics of gartersnake fitness suggest that fCM concentrations are not appropriate indicators of mean population fitness, and thus of habitat suitability for gartersnakes.

The differences in fitness we documented between field and forest habitats are likely to be caused by thermal quality; field provides a more suitable thermal habitat than forest in our study area (W. D. Halliday and G. Blouin-Demers unpublished observations). Yet fCM concentrations in our study did not differ between snakes living in field and in forest habitats, and also did not vary with body temperature. Stress hormones exhibit variable relationships with temperature across different taxa. Fish have demonstrated a consistently positive relationship between cortisol and temperature (e.g. Barton and Schreck, 1987; Davis *et al*., 2001; O'Connor *et al*., 2011), whereas reptiles have exhibited positive (e.g. Girling and Cree, 1995; Tyrell and Cree, 1998; Schramm *et al*., 1999; Cree *et al*., 2003; Woodley *et al*., 2003), negative (Dupoué *et al*., 2013) or no relationship (Dunlap and Wingfield, 1995) between corticosterone and temperature. It is clear that habitat alone does not lead to differences in fCM concentrations, even habitats differing strongly in thermal quality, at least in part because body temperature does not appear to affect fCM concentrations in common gartersnakes.

In animals, important differences between habitats include energetic (food or thermal quality) and structural differences (e.g. presence of cover or nesting sites), which can have different physiological consequences. Structural differences between habitats could lead to differences in predation risk, and animals could then experience increased stress hormones in the riskier habitat (Boonstra *et al*., 1998). Habitats with different levels of disturbance (human or natural) can also lead to differences in stress hormones (Homan *et al*., 2003). Habitats differing in energetic potential might cause differences in physiological metrics that are related to energetics, such as field metabolic rate. Given that metabolism is directly affected by temperature in ectotherms (Gillooly *et al*., 2001; Dubois *et al*., 2009), the thermal quality of habitats is likely to be an important driver of metabolic differences. Understanding what ecological factors differ between habitats, and then determining how these differences might affect fitness, is the first step in accurately measuring habitat suitability. Physiological metrics are likely to be useful only when they are directly linked to differences in habitat suitability.

In summary, our measures of reproductive output and of growth rate confirm that an increased abundance of gartersnakes in field habitat does indeed reflect higher habitat suitability, whereas our measure of stress hormones in snakes was invariant between habitats. Therefore, researchers should use multiple metrics of fitness when measuring habitat suitability to confirm inferences based on presence or abundance. Researchers planning to use physiological metrics of habitat suitability should carefully select a metric that is likely to reflect fitness differences between habitats, and it would be prudent to assess the usefulness of such physiological metrics in pilot studies.

## Supplementary material

Supplementary material is available at *Conservation Physiology* online.

## Funding

This project was funded by the University of Ottawa, a Natural Science and Engineering Research Council (NSERC) of Canada Post-Graduate Scholarship to W.D.H., and NSERC Discovery Grants to K.M.G. and G.B.-D.

## Supplementary Material

Supplementary Data
