# Supplementary material for: Faecal corticosterone metabolite concentrations are not a good predictor of habitat suitability for common gartersnakes
Source: Conserv Physiol. 2015 Oct 20;3(1):cov047. doi: 10.1093/conphys/cov047 (PMC4778491; doi:10.1093/conphys/cov047)
Supplement: Supplementary Data [file cov047supp.zip › cov047supp.docx]

Appendix 1 - Validation of the use of faecal corticosterone metabolite levels to estimate circulating corticosterone levels

The goal of this experiment was to test whether changes in faecal corticosterone metabolite (fCM) levels match changes in circulating (plasma) corticosterone levels, which would thus confirm that fCM levels can be used as a proxy for circulating corticosterone levels. We collected eight female Common Gartersnakes (*Thamnophis sirtalis*) in fields and wetlands near Ottawa, Ontario, and transported them back to our animal care facility at the University of Ottawa. We housed each female alone in a small transparent plastic container (31 × 17 × 10 cm) with newspaper as a substrate, an artificial shelter, *ad libitum* water, and a hotspot provided by Flexwatt tape under one end of the container. We placed all containers in an environmental chamber set to a 14:10 hour light:dark cycle, with temperatures reaching 25°C during the day, and dropping to 10°C during the night. This light and temperature cycle represents average seasonal levels. When individuals were not being used for an experimental treatment, we fed them two large earthworms every two days.

To assess whether fCM levels mirror plasma corticosterone levels, we collected a faecal pellet and a blood sample from each individual. We first collected a blood sample (200 µl) from the caudal vein using a syringe fitted with a 28-gauge needle; blood was collected within 3 minutes of handling each snake. We then collected a faecal pellet by gently palpating the abdomen towards the cloaca. Following the collection of blood and faecal samples, we fed each snake two large earthworms that had each been injected with 30 μg of corticosterone mixed in 1 ml of DMSO. We left the snakes undisturbed for 24 hours, and then again collected blood and faecal samples. We maintained the ambient temperature in the environmental chamber at 25°C during this period to speed up digestion, which allowed us to reliably collect a faecal sample and a blood sample simultaneously after 24 hours. We centrifuged blood samples immediately after collection at 210 *g* for 10 minutes, removed the plasma from the samples, and flash froze the plasma in liquid nitrogen. We placed the faecal pellets on ice upon collection and stored samples at -80°C until extraction and/or analysis (see main text for extraction method and procedure). We followed the same extraction and RIA procedures for faecal samples as described above. For plasma samples, we followed the directions of the RIA kit (10 μl of plasma sample in 2 ml of steroid diluent).

We compared plasma corticosterone levels and fCM levels of the snakes before and after they ate corticosterone-injected worms using linear mixed effects models, with log_10_-transformed corticosterone level as the dependent variable, sample type (faecal or plasma), sample time (before or after corticosterone treatment), and their interaction as fixed effects, and snake ID as a random effect. Only six of the eight snakes ate the corticosterone-injected earthworms, therefore the analysis was based on these six snakes.

Plasma corticosterone concentrations did not significantly differ from fCM levels (*t_1,17_* = 0.26, *p* = 0.80), but both concentrations increased following the corticosterone treatment (pre-fCM: 108.8 ± 44.9 ng ml^-1^; pre-plasma: 63.3 ± 18.4 ng ml^-1^ ; post-fCM: 643.2 ± 121.2 ng ml^-1^; post-plasma: 1385.3 ± 227.4 ng ml^-1^; *t_1,17_* = 8.84, *p* < 0.01; Table A1; Figure A1). Snakes that ate corticosterone-injected worms had elevated plasma corticosterone levels, and these elevated plasma corticosterone levels led to an increase in fCM levels. Therefore, fCM levels can be a useful metric of plasma corticosterone levels.

Table A1. Model selection and final model output for linear mixed effects models examining the effect of sample type (faecal or plasma) and corticosterone treatment (before or after) on log_10_ corticosterone levels in six female Common Gartersnakes (*Thamnophis sirtalis*). *k* is the number of parameters in the model, AICc is the bias-corrected Akaike’s information criteria value for the model, and ΔAICc is the difference between a model and the model with the lowest AICc.

| Model | | | *k* | AICc | | ΔAICc |
| --- | --- | --- | --- | --- | --- | --- |
| Corticosterone = Treatment | | | 4 | 25.10 | | 0.00 |
| Corticosterone = Type + Treatment + Type : Treatment | | | 6 | 28.83 | | 3.73 |
| Corticosterone = Type + Treatment | | | 5 | 29.67 | | 4.57 |
| Corticosterone = Type | | | 4 | 58.36 | | 33.26 |
| Parameter | Estimate | S.E. | | | *t* | *p* |
| Intercept | 2.93 | 0.09 | | | 32.29 | < 0.01 |
| Treatment (Pre) | -1.14 | 0.13 | | | 8.84 | < 0.01 |

Figure A1. Boxplot of corticosterone levels in six female Common Gartersnakes (*Thamnophis sirtalis*), measured in faeces and plasma, before and after consuming corticosterone-injected earthworms. The box represents the interquartile range, the line within the box is the median, and the whiskers represent the minimum and maximum values. ’Plasma’ represents circulating corticosterone concentration, whereas ‘faeces’ represents faecal corticosterone metabolite levels.

Figure A2. Serial dilution of extracted faecal corticosterone metabolite samples of six Common Gartersnakes (*Thamnophis sirtalis*) gave a displacement curve parallel to the corticosterone standard curve (sample slope 95% CI: -1.17 to 1.05; standard slope 95% CI: -0.89 to -0.50; *t_1,8_* = 1.23, *p* = 0.26).
